# O Coração de Pacientes Pediátricos com COVID-19: Novos *Insights* a Partir de um Estudo Ecocardiográfico Sistemático em um Hospital Terciário no Brasil

**DOI:** 10.36660/abc.20200920

**Published:** 2021-06-10

**Authors:** Maria de Fátima Rodrigues Diniz, Maira Freire Cardoso, Karen Saori Shiraishi Sawamura, Carolina Rocha Brito Menezes, Alessandro Cavalcanti Lianza, Maria Fernanda Badue Pereira, Nadia Litvinov, Juliana Ferreira Ferranti, Silvana Forsait, Andreia Watanabe, Sylvia Costa Lima Farhat, Nadia Emi Aikawa, Lucia Maria Arruda Campos, Artur Figueiredo Delgado, Magda Carneiro-Sampaio, Werther Brunow de Carvalho, Clovis Artur Silva, Gabriela Nunes Leal

**Affiliations:** 1 Universidade de São Paulo Instituto da Criança São Paulo SP Brasil Universidade de São Paulo Instituto da Criança, São Paulo, SP - Brasil; 2 Hospital do Coração São Paulo SP Brasil Hospital do Coração, São Paulo, SP - Brasil; 3 Hospital Israelita Albert Einstein São Paulo SP Brasil Hospital Israelita Albert Einstein, São Paulo, SP - Brasil; 4 Universidade de São Paulo Hospital das Clínicas Faculdade de Medicina São Paulo SP Brasil Universidade de São Paulo Hospital das Clínicas - Faculdade de Medicina,São Paulo, SP - Brasil; 5 Universidade de São Paulo Faculdade de Medicina São Paulo SP Brasil Universidade de São Paulo - Faculdade de Medicina, São Paulo, SP - Brasil; 6 Hospital Sírio-Libanês São Paulo SP Brasil Hospital Sírio-Libanês, São Paulo, SP - Brasil

**Keywords:** COVID-19, Pandemia, Betacoronavírus, Biomarcadores, Inflamação, Criança, Insuficiência Cardíaca, Ecocardiografia/métodos

## Abstract

**Fundamento:**

A pandemia da COVID-19 representa uma enorme carga para o sistema de saúde do mundo. Apesar de pacientes pediátricos terem sido relativamente poupados em comparação a adultos, estudos recentes mostraram um número crescente de pacientes críticos com Síndrome Inflamatória Multisistêmica Pediátrica (SIM-P) com disfunção cardiovascular importante. No entanto, pouco se conhece a respeito da relação entre anormalidades cardíacas e biomarcadores inflamatórios e de coagulação.

**Objetivos:**

Investigar anormalidades ecocardiográficas em pacientes pediátricos com COVID-19 admitidos em um hospital terciário.

**Métodos:**

Este foi um estudo longitudinal retrospectivo, baseado na revisão de prontuários médicos e ecocardiogramas de pacientes (0-19 anos) admitidos em um hospital terciário entre 30 de março e 30 de junho de 2020. Para a análise estatística, o nível de significância foi estabelecido em 5% (p<0,05).

**Resultados:**

Foram incluídos 48 pacientes, 73% com doenças pré-existentes, 20 (41,7%) com SIM-P. A idade mediana foi 7,5 (0-18,6) anos; 27 (56,2%) eram do sexo masculino. A duração mediana de internação foi 15,4 (2-92) dias e sete (14,6%) pacientes morreram. Um total de 70 ecocardiografias foram realizadas, 66,7% submeteram-se ao exame somente uma vez, e 33,3% várias vezes. Vinte e três (48%) pacientes apresentaram anormalidades no ecocardiograma: oito (16.6%) disfunção sistólica do ventrículo esquerdo, seis (12.5%) disfunção sistólica do ventrículo direito, e 12 (25%) dilatação da artéria coronária (Z-score>+2,5). Anormalidades ecocardiográficas foram significativamente associadas com SIM-P, admissão na unidade de terapia intensiva pediátrica, suporte ventilatório/vasoativo, e morte ( *p* <0,05). Níveis significativamente mais altos de d-dímero (ng/mL) foram detectados em pacientes com disfunção ventricular esquerda [16733(4157-115668) vs. 2406.5(190-95040)], disfunção ventricular direita [25769(3422-115668) vs. 2803.5(190-95040)] e dilatação da artéria coronária [9652.5(921-115668) vs. 2724(190- 95040)] (p<0,05).

**Conclusão:**

Anormalidades ecocardiográficas eram frequentes nos pacientes pediátricos com COVID-19 e associadas com piores desfechos clínicos. Exacerbação das vias de inflamação e coagulação pode exercer um importante papel na lesão cardiovascular nesses pacientes.

## Introdução

A pandemia da doença por coronavírus-2019 (COVID-19) representa uma enorme carga para o sistema de saúde em todo o mundo. Em sua apresentação mais grave, a COVID-19 é uma doença sistêmica caracterizada por hiperinflamação, tempestade de citocinas, e níveis aumentados de marcadores de lesão do miocárdio.^1^ O envolvimento cardíaco parece ser uma característica importante da doença em adultos, acometendo 20 a 30% dos pacientes internados e contribuindo para 40% das mortes.^2^ Apesar de as crianças terem sido relativamente poupadas quando comparadas aos adultos, estudos recentes mostraram um número crescente de pacientes críticos com Síndrome Infamatória Multissistêmica Pediátrica (SIM-P), acompanhada por uma grave disfunção cardiovascular.^3^ Disfunção ventricular, efusão pericárdica, regurgitação valvar, e inflamação da artéria coronária foram registrados em muitas séries de casos. Um fenótipo do tipo Kawasaki também foi descrito em alguns pacientes com SIM-P, apesar de a literatura recente sugerir que esses são diferentes tipos de doenças com características clínicas que se sobrepõem. Até o presente, a SIM-P ocorre predominantemente em crianças mais velhas, com idade mediana de 9-10 anos, ao passo que a doença de Kawasaki afeta tipicamente crianças menores de 5 anos. Choque cardiovascular, raramente encontrado na doença de Kawasaki, é uma característica marcante da SIM-P.^3^

Contudo, a real incidência de anormalidades cardíacas em pacientes pediátricos com COVID-19 e sua importância para desfechos clínicos precisam ainda ser determinados. Pouco se sabe sobre a relação entre anormalidades cardíacas, e marcadores inflamatórios e de coagulação nesse grupo.^4^ Consequentemente, existe uma necessidade urgente de se melhor compreender as interações entre COVID-19 e o coração na população pediátrica.

O presente estudo teve como objetivo investigar anormalidades ecocardiográficas de crianças com COVID-19 admitidos em um hospital terciário de São Paulo, o epicentro da pandemia da COVID-19 no Brasil. Possíveis associações de dados clínicos e laboratoriais com achados ecocardiográficos também foram exploradas.

## Métodos

### Delineamento e população do estudo

Este é um estudo longitudinal retrospectivo, baseado na revisão de prontuários médicos e laudos de ecocardiograma de crianças e adolescentes (0-19 anos) admitidos na enfermaria de pediatria e na unidade de terapia intensiva por COVID-19, entre 30 de março e 30 de junho de 2020. Foram incluídos pacientes com e sem SIM-P, de acordo com a classificação da Organização Mundial da Saúde (OMS).^5^ O critério de exclusão foi ausência de ecocardiogramas durante o período de acompanhamento.

### Parâmetros clínicos, laboratoriais e terapêuticos

Os prontuários médicos eletrônicos dos pacientes foram cuidadosamente revisados quanto aos dados clínicos, laboratoriais e terapêuticos. Doenças pré-existentes e ecocardiogramas prévios também foram registrados. O estudo foi aprovado pelo comitê de ética em pesquisa da instituição.

Os pacientes foram classificados como apresentando SIM-P se preenchessem os seguintes critérios:

1. Crianças e adolescentes (0-19 anos) com febre por três dias ou mais.
2. E pelo menos dois dos seguintes quadros:
  - *Rash* , conjuntivite bilateral não purulenta, ou sinais de inflamação mucocutânea (oral, mãos ou pés)
  - Hipotensão ou choque
  - Características de disfunção do miocárdio, pericardite, valvulite, ou anormalidades coronárias (incluindo achados ecocardiográficos ou enzimas cardíacas elevadas)
  - Evidência de coagulopatia (níveis elevados de d-dímero, tempo de protrombina, tempo de tromboplastina parcialmente ativada)
  - Problemas gastrointestinais agudos (diarreia, vômito, ou dor abdominal).
3. E: níveis elevados de marcadores inflamatórios, tais como taxa de sedimentação de eritrócitos (TSE), proteína C- reativa, e procalcitonina.
4. E: sem outra evidência de causa bacteriana de inflamação
5. E: infecção confirmada de síndrome respiratória aguda grave 2 (SARS-CoV-2) por reação em cadeia de polimerase em tempo real (RT-PCR) e/ou serologia, ou contato provável com pacientes com COVID-19.

O exame de RT-PCR em amostras respiratórias foi realizado para detectar RNA de SARS-CoV-2. Testes sorológicos incluíram dois métodos diferentes durante a pandemia da COVID-19: teste imunocromatográfico para detecção de anticorpo IgM/IgG específico para SARS-Cov-2 e ensaio de imunoabsorção enzimática (ELISA) para detecção de anticorpos IgG.^6^

Pacientes com SIM-P e sem SIM-P foram comparados quanto a idade, sexo, sinais e sintomas clínicos na apresentação, frequência de anormalidades ecocardiográficas, infecção por SARS-CoV-2 confirmada, e morte. Os seguintes dados laboratoriais foram comparados: frequência de anemia, linfocitopenia e trombocitopenia, evidência de coagulopatia, pico sérico de d-dímero, PCR, ferritina, troponina, e creatinina quinase MB. Pró-peptídeo natriurético cerebral (pro-BNP), procalcitonina e fibrinogênio não foram incluídos na análise, uma vez que esses biomarcadores não foram avaliados rotineiramente em todos os pacientes.

Anemia foi definida como hematócrito igual ou menor que percentil 2,5 para idade, raça e sexo;^7^ linfocitopenia como uma contagem de linfócitos menor que 4500/mm^3^ em crianças abaixo de oito meses de idade, e 1500/mm^3^ para crianças acima dessa idade;^8^ e trombocitopenia foi definida como contagem de plaquetas menor que 1000 000/microL.^9^

### Ecocardiografia

Todos os exames de ecocardiografia foram realizados por dois cardiologistas pediátricos experientes, de acordo com as diretrizes da Sociedade Americana de Ecocardiografia (ASE, *American Society of Echocardiography* ).^10^ As análises incluíram o modo M e o modo bidimensional (2D), além do exame padrão de Doppler colorido. O equipamento utilizado foi um aparelho Philips Affinity 70, CX50 e um ultrassom compacto Innosight, com transdutores multifrequência (S5-1 e S8-3). Os exames de ecocardiografia também seguiram as recomendações da ASE sobre proteção dos pacientes e serviços de ecocardiografia durante a pandemia da COVID-19 (Statement on Protection of Patients and Echocardiography Service Providers During the 2019 Novel Coronavirus Outbreak).^11^ Uma vez que um dos aparelhos utilizados em nossa instituição durante a pandemia da COVID-19 foi originalmente desenhado como um ultrassom “point-of-care” (Philips Innosight), ou seja, a ser usado à beira do leito ou no local de atendimento do paciente, não foi possível obter fração de ejeção bidimensional do ventrículo esquerdo (método de Simpson) em todos os escaneamentos. Por isso, escolhemos a fração de ejeção obtida pelo modo-M (método Teichholz), apesar de o método de Simpson ser sabidamente mais preciso.^10^ Disfunção sistólica do ventrículo esquerdo foi definida como uma fração de ejeção de ventrículo esquerdo (FEVE) < 55%, e foi considerada leve se a FEVE fosse ≥45% e < 55%, moderada se a FEVE fosse ≥ 30% e <45%, e grave se a FEVE fosse < 30%.^10^

A função sistólica do ventrículo direito (VD) foi avaliada pela excursão sistólica do plano anular tricúspide (TAPSE). Disfunção sistólica do VD foi detectada quando o z-score da TAPSE fosse menor que -2.^12^

As artérias coronárias foram avaliadas de acordo com as diretrizes da *American Heart Association* para o diagnóstico, tratamento e manejo em longo prazo da doença de Kawasaki.^13^ Dilatação foi detectada quando o z-score do diâmetro do lúmen interno da artéria coronária fosse maior que +2,5.^14^ Um z-score entre +2,5 e +5 foi usado para definir pequenos aneurismas, z-score entre +5 e +10 médios aneurismas, e ≥+10, aneurismas gigantes. Outros sinais ecocardiográficos frequentemente descritos na inflamação da artéria coronária, como realce perivascular e ausência de afilamento, também foram registrados.^13^

A pressão sistólica da artéria pulmonar (PSAP) foi estimada pela regurgitação tricúspide; hipertensão pulmonar (HP) foi diagnosticada quando a pressão sistólica da artéria pulmonar foi maior que 35 mmHg. HP leve foi diagnosticada quando a PSAP era > 35mmHg e ≤ 45 mmHg, moderada quando a PSAP era > 45mmHg e ≤ 50 mmHg, e grave quando a PSAP era > 50 mmHg.^15^

Presença de efusão pericárdica também foi descrita, bem como de sinais eventuais de tamponamento cardíaco.

Os pacientes foram divididos de acordo com a presença ou ausência de anormalidades ecocardiográficas, e comparados quanto à idade, sexo, presença de SIM-P, admissão na unidade de terapia intensiva (UTI) pediátrica, presença de disfunção de múltiplos órgãos, suporte ventilatório/vasoativo, tratamento de substituição renal, uso de imunoglobulina endovenosa, corticosteroides, ácido acetilsalicílico e heparina de baixo peso molecular, tempo de internação, e morte.

As imagens foram adquiridas digitalmente, e a variabilidade intraobservador e entre observadores para FEVE, TAPSE, e diâmetro das artérias coronárias foi avaliada. O mesmo examinador repetiu a análise de 10 exames selecionados aleatoriamente. Um segundo observador (CRB), que não conhecia os resultados anteriores e a condição clínica do paciente, também realizou as medidas ecocardiográficas, de maneira *offline* .

### Análise estatística

A análise estatística foi realizada usando o programa IBM SPSS Statistics 22. Os dados categóricos foram descritos como porcentagens, e os dados contínuos como média (desvio padrão, DP) ou mediana (intervalo). O teste exato de Fisher foi usado para comparar dados categóricos. O teste de Kolmogorov e Smirnov foi usado para verificar se os dados tinham uma distribuição normal. O teste t de Student não pareado foi usado para avaliar variáveis contínuas com distribuição normal, e o teste de Mann-Whitney usado para avaliar variáveis contínuas sem distribuição normal. O nível de significância foi estabelecido em 5% (p<0,05). Variabilidade intraobservador e entre observadores para as medidas ecocardiográficas foi avaliada usando o gráfico de Bland-Altman e coeficiente de correlação intraclasse (CCI), e um CCI > 0,8 foi definido como uma boa correlação.

## Resultados

### Apresentação clínica

Quarenta e oito pacientes pediátricos foram hospitalizados por COVID-19 durante o período do estudo. A idade mediana foi 7,5 (0 – 18,6) anos; 21 (43,8%) eram do sexo feminino. O tempo mediano de internação foi 15,4 (2 - 92) dias. Até o final do estudo, 33 (68,7%) pacientes receberam alta com sucesso, oito (16,7%) ainda se encontravam na enfermaria ou na UTI pediátrica, e sete (14,6%) pacientes morreram. Todos os óbitos ocorreram no grupo SIM-P. Não se observou diferença estatisticamente significativa entre sobreviventes e pacientes que foram a óbito quanto ao uso de corticosteroide, imunoglobulina endovenosa ou heparina de baixo peso molecular.

Vinte (41,7%) pacientes preencheram os critérios da OMS para SIM-P e 28 (58,3%) não. Entre os pacientes com SIM-P, 11 (55%) apresentaram infecção por SARS-CoV-2 confirmada por RT-PCR e/ou sorologia, e nove (45%) não. Todos os nove pacientes com SIM-P sem infecção por SARS-CoV-2 confirmada tiveram contato próximo com pacientes com COVID-19 nas quatro últimas semanas anteriores aos sintomas. Cinco dos nove pacientes também apresentaram achados típicos na tomografia computadorizada de tórax infectados (opacidades em vidro fosco circundada por anelo de consolidação (sinal do halo).

Nos pacientes sem SIM-P (n=28), a infecção por SARS-CoV-2 foi confirmada por PCR e/ou sorologia.

SIM-P foi associada com convulsões, choque, evidência de coagulopatia, anormalidades no ecocardiograma, e morte. Picos significativamente mais altos de d-dímero, PCR e troponina séricos foram detectados em pacientes com SIM-P (p<0,05). A incidência de sintomas respiratórios e gastrointestinais foi similar entre pacientes com e sem SIM-P (p>0,05). Ainda, não foi observada diferença na frequência de anemia, linfocitopenia, trombocitopenia, ou doenças pré-existente entre os dois grupos de pacientes (p>0,05). Somente um paciente apresentou sinais de inflamação mucocutânea. Nenhum paciente preencheu os critérios diagnósticos para doença de Kawasaki ( Tabela 1 ).

**Tabela 1:** – Dados demográficos, clínicos, e laboratoriais de pacientes pediátricos com COVID-19 com e sem Síndrome Inflamatória Multissistêmica Pediátrica (SIM-P), segundo critérios da Organização Mundial da Saúde

Dados demográficos, clínicos e laboratoriais	MIS-c ( *n* = 20)	Sem MIS-c ( *n* = 28)	p
Idade (anos)	8,4 (0,1-16,4)	6,7 (0 – 18,6)	0,33
Sexo (masculino)	10 (50%)	17 (60,7%)	0,56
Doenças pré-existentes	15 (75%)	20 (71,4%)	1
Sintomas respiratórios	10 (50%)	14 (50%)	1
Sintomas gastrointestinais	6 (30%)	7 (25%)	0,75
*Rash* / Conjuntivite bilateral não purulenta /sinais de inflamação mucocutânea	1 (5%)	0 (0%)	0,41
Convulsões	5 (25%)	0 (0%)	0,009
Choque	12 (60%)	0 (0%)	<0,0001
Evidência de coagulopatia (↑TP, ↑TTP, ↑D-dímero)	20 (100%)	18 (64,3%)	0,0028
Anemia*	14 (70%)	21 (75%)	0,75
Trombocitopenia**	4 (20%)	7 (25%)	0,74
Linfocitopenia***	8 (40%)	14 (50%)	0,56
Anormalidades ecocardiográficas	19 (95%)	4 (14,3%)	<0,0001
D-dímeros (ng/ml)****	9652,5 (921 - 115668)	1722 (190 - 95040)	0,0003
Proteína C reativa (mg/L)****	119,6 (0,38 - 447,7)	14,6 (0,30 – 324)	0,0046
Ferritina (ng/ml)****	1159 (58-35967)	655 (25-2567)	0,07
Troponina (ng/L)****	25 (9-385)	16 (3-1050)	0,028
Creatina quinase MB (ng/ml)****	1,78 (0,3-30)	1,65 (0,18-28,9)	1
Morte	7 (35%)	0 (0%)	0,001
Infecção por Sars-CoV-2 confirmada (RT-PCR/sorologia)	11 (55%)	28 (100%)	0,0001

Valores expressos em n (%) ou mediana (intervalo). Teste exato de Fisher foi usado para comparar dados categóricos. O teste de Mann-Whitney usado para comparar variáveis contínuas sem distribuição normal. *Hematócrito ≤ percentil 2,5 para idade, sexo, e raça na admissão; ***Contagem de linfócitos < 4500/mm^3^ na admissão em crianças com idade abaixo de 8 meses e < 1500/mm^3^ em crianças acima dessa idade; ****Valores correspondem ao valor sérico mais alto obtido de cada paciente; TP: tempo de protrombina; TTP: tempo de tromboplastina parcialmente ativada

Doenças pré-existentes foram detectadas em 35 (73%) pacientes: imunossupressão em 26 (54,2%), doenças malignas em 14 (40%), doença renal crônica em nove (25,7%), neuropatia crônica em oito (22,8%), doença cardíaca adquirida ou congênita em cinco (14,2%), pneumopatia crônica em cinco (14,2%), hepatopatia em quatro (11,4%), síndromes dismórficas em três (6,3%), distrofia muscular de Duchenne em um (2.8%), lúpus eritematoso sistêmico juvenil em um (2,8%), cirurgia ortopédica prévia em um (2,8%), cirurgia ginecológica prévia em um (2,8%), transplante cardíaco em um (2,8%). Dois (4,2%) pacientes eram neonatos de mães com COVID-19, e um deles também era prematuro. Entre os sete pacientes que foram a óbito, três eram pacientes oncológicos (dois com tumores sólidos e um com leucemia), um teve imunodeficiência primária, um teve síndrome de Edwards com doença cardíaca congênita, e dois eram sadios.

### Avaliação ecocardiográfica

Os 48 pacientes realizaram pelo menos um exame de ecocardiografia durante a internação. Trinta e dois (66,7%) realizaram somente um exame e 16 (33,3%) submeteram-se ao exame várias vezes. Um total de 70 exames foram realizados durante o período do estudo. Todos os pacientes com doenças pré-existentes já eram acompanhados em nossa instituição e apresentavam laudos de ecocardiograma em seus prontuários médicos. Cinco (14,2%) pacientes apresentavam anormalidades no ecocardiograma previamente: um com pequeno defeito do septo ventricular e válvula aórtica bicúspide (síndrome de Edwards), um com pequeno defeito residual do septo ventricular e discreta coarctação da aorta, um com uma massa ecogênica invadindo a veia cava inferior (tumor adrenal), um com discreta hipertrofia do ventrículo esquerdo secundária à doença renal crônica, e um com disfunção sistólica moderada no ventrículo esquerdo secundária à quimioterapia (sarcoma).

Vinte e três (48%) pacientes apresentaram anormalidades ecocardiográficas, e 19 (39,6%) deles apresentaram novos achados ecocardiográficos potencialmente associados com COVID-19: disfunção do VD e esquerdo, dilatação da artéria coronária, hipertensão pulmonar, e efusão pericárdica. Vale ressaltar que somente um paciente com anormalidades ecocardiográficas prévias apresentou com novos achados ecocardiográficos: disfunção sistólica do ventrículo esquerdo secundária à quimioterapia progrediu de moderada à grave, e dilatação da artéria coronária também foi detectada.

Anormalidades ecocardiográficas foram associadas a SIM-P, admissão da UTI pediátrica, disfunção de múltiplos órgãos, suporte ventilatório e vasoativo, uso de imunoglobulina endovenosa, corticosteroide, ácido acetilsalicílico e heparina de baixo peso molecular, e morte ( Tabela 2 ). Pacientes com anormalidades ecocardiográficas também apresentaram maior tempo de hospitalização.

**Tabela 2:** – Dados demográficos e desfechos clínicos segundo presença ou ausência de anormalidades ecocardiográficas

Dados demográficos, estratégias terapêuticas e desfechos clínicos	Anormalidades ecocardiográficas		
	Presentes ( *n* = 23)	Ausentes ( *n* = 25)	p
Idade (anos)	7,8 (0,1-16,4)	6,4 (0-18,6)	0,87
Sexo (masculino)	11 (47,8%)	16 (64%)	0,38
SIM-P segundo critérios da OMS	19 (82,6%)	1 (4%)	<0,0001
Unidade de terapia intensiva pediátrica	15 (65,2%)	5 (20%)	0,003
Síndrome de disfunção de múltiplos órgãos	8 (34,8%)	0 (0%)	0,0013
Sistema respiratório	6 (26%)	0 (0%)	
Sistema cardiovascular	6 (26%)	0 (0%)	
Sistema renal	5 (21,7%)	0 (0%)	
Sistema hepático	2 (8,7%)	0 (0%)	
Sistema neurológico	4 (17,4%)	0 (0%)	
Sistema hematológico	4 (17,4%)	0 (0%)	
Suporte ventilatório	15 (65,2%)	7 (28%)	0,02
Oxigênio por cateter nasal	8 (34,8%)	3 (12%)	
Máscara de Venturi	3 (13%)	1 (4%)	
Máscara sem *rebreather*	0 (0%)	1 (4%)	
Oxigenoterapia de alto fluxo	6 (26%)	1 (4%)	
Ventilação não invasiva	5 (21,7%)	1 (4%)	
Ventilação mecânica convencional	10 (43,5%)	3 (12%)	
Ventilação de alta frequência	1 (4,3%)	0 (0%)	
Suporte com drogas vasoativas	10 (43,5%)	1 (4%)	0,0015
Epinefrina	4 (17,4%)	0 (0%)	
Norepinefrina	10 (43,5%)	1 (4%)	
Vasopressina	2 (8,7%)	0 (0%)	
Milrinona	5 (21,7%)	1 (4%)	
Dobutamina	3 (13%)	0 (0%)	
Tratamento de substituição renal	5 (21,7%)	2 (8%)	0,23
Diálise peritoneal	0 (0%)	2 (8%)	
Hemodiálise convencional	1(4,3%)	0 (0%)	
Hemodiálise prolongada	1 (4,3%)	0 (0%)	
Hemodiálise contínua	3 (13%)	0 (0%)	
Imunoglobulina endovenosa	14 (60,8%)	0 (0%)	<0,0001
Corticosteroides	4 (17,4%)	0 (0%)	0,04
Ácido acetilsalicílico	9 (39%)	0 (0%)	0,0005
Heparina de baixo peso molecular	9 (39,1%)	1 (4%)	0,0038
Tempo de hospitalização (dias)	23 (2-92)	8,3 (2-26)	0,0074
Mortes	6 (26%)	1 (4%)	0,04

*Valores expressos em n (%) ou mediana (intervalo). Teste exato de Fisher foi usado para comparar os dados categóricos. O teste de Mann-Whitney usado para comparar variáveis contínuas sem distribuição normal.*

Dez (20,8%) dos 48 pacientes receberam heparina de baixo peso molecular durante a internação, somente um paciente sem anormalidade ecocardiográfica. Terapia de anticoagulação foi introduzida em dois pacientes: um com z-score da artéria coronária esquerda de +10, e um com disfunção sistólica grave do ventrículo esquerdo e trombose da veia subclávia. Os demais oito pacientes receberam heparina de baixo peso molecular profilática devido ao tempo prolongado de hospitalização, doenças malignas concomitantes e uso prolongado de cateter.

A disfunção sistólica do ventrículo esquerdo foi detectada em oito (16,6%) pacientes: seis com disfunção leve, um com disfunção moderada, e um com disfunção grave. Hipocinesia global do ventrículo esquerdo foi detectada em todos os pacientes, exceto um, que apresentou acinesia apical, sugestivo de síndrome de Takotsubo. Quatro pacientes com disfunção do ventrículo esquerdo também apresentaram anormalidades das artérias coronárias. Pacientes com disfunção sistólica do ventrículo esquerdo apresentaram picos significativamente mais altos de d-dímero, PCR, ferritina e troponina ( Tabela 3 ). Cinco pacientes com disfunção do ventrículo esquerdo receberam imunoglobulina endovenosa e somente um recebeu corticosteroides. Nenhum paciente recebeu bloqueadores de interleucina. Cinco pacientes apresentaram melhora da função sistólica ventricular esquerda durante o acompanhamento.

**Tabela 3:** – Perfil laboratorial dos pacientes de acordo com anormalidades ecocardiográficas detectadas no estudo

Dados laboratoriais*	Disfunção sistólica do ventrículo esquerdo			Disfunção sistólica do ventrículo direito			Anormalidades da artéria coronária		
	Presente (n = 8)	Ausente (n = 40)	p	Presente (n = 6)	Ausente (n = 42)	p	Presente (n =12)	Ausente (n = 36)	p
D-dímero (ng/ml)	16733 (4157 -115668)	2406,5 (190 - 95040)	0,0015	25769 (3422 - 115668)	2803,5 (190 - 95040)	0,037	9652,5 (921 - 115668)	2724 (190 - 95040)	0,04
PCR (mg/L)	303,16 (30 - 423)	35,9 (0,3 - 447,7)	0,0017	113,95 (2 - 407,21)	53,95 (0,3 - 447,70)	0,46	109,9 (0,38 – 423)	33,75 (0,38 - 447)	0,10
Ferritina (ng/ml)	3734 (839 - 35967)	499 (25 - 8000)	0,0026	1301 (123 - 35967)	663 (25 - 8000)	0,18	389,50 (58 - 35967)	790 (25 - 8000)	0,8
Troponina (ng/L)	88 (20 - 342)	16 (3 - 1050)	0,0018	108,5 (3 - 385)	17 (3 - 1050)	0,04	19,5 (9 - 125)	19 (3 - 1050)	0,57
CK-MB (ng/ml)	2,2 (0,7 - 28)	1,6 (0,18 - 30,7)	0,62	4 (0,18 - 30,7)	1,6 (0,3 -28,9)	0,58	1,78 (0,3 -18,2)	1,65 (0,18 – 30,7)	0,9

Teste de Mann-Whitney foi usado para comparar variáveis contínuas sem distribuição normal. *Valores correspondem aos valores séricos mais altos obtidos de cada paciente, e são expressos em mediana (intervalo). PCR: proteína C-reativa; CK-MB: creatina quinase-MB.

Seis (12,5%) pacientes apresentaram disfunção sistólica do VD. Esses pacientes apresentaram picos significativamente mais altos de d-dímero e troponina ( Tabela 3 ). Dois pacientes apresentaram HP leve, e três também apresentaram disfunção sistólica do ventrículo esquerdo. Melhora da função sistólica do VD foi observada em três pacientes durante o acompanhamento.

**Figura 1:**
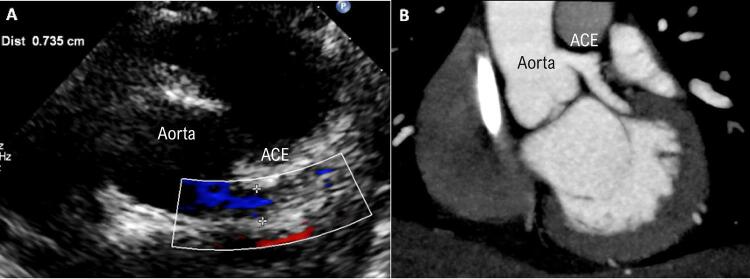
– A) Ecocardiograma mostrando dilatação da artéria coronária esquerda (ACE) em uma adolescente de 15 anos; B) Tomografia computadorizada do mesmo paciente.

Anormalidades da artéria coronária foram detectadas em 12 (25%) pacientes, e a maioria exibiu ectasia leve, exceto um adolescente (15 anos de idade) com z-score da artéria coronária esquerda de +10 ( Figura 1 ). Além de dilatação, seis pacientes apresentaram realce perivascular. Dilatação da artéria coronária esquerda encontrava foi detectada em 11 pacientes, com z-score mediano de +4 (+2,8 - +10); dilatação da artéria descendente anterior esquerda (ADE) e, seis, com um z-score mediano de +4 (+3.6 - +4.2); dilatação da artéria circunflexa em três, com um z-score mediano de +4.6 (+3.9 - +5); e dilatação da artéria coronária direita (ACD), com um z-score mediano de +3.3 (+2.6 - +4.3). Pacientes com anormalidades da artéria coronária apresentaram picos significativamente mais altos de d-dímero ( Tabela 3 ). Em quatro pacientes, anormalidades da artéria coronária não estavam presentes na primeira avaliação ecocardiográfica, e foram detectadas em exames subsequentes.

Onze (91,7%) dos 12 pacientes com dilatação da artéria coronária receberam imunoglobulina endovenosa. Em um paciente, a dilatação da artéria coronária foi detectada tardiamente, após estar afebril por mais de uma semana. Nove (75%) dos 12 pacientes com dilatação da artéria coronária receberam ácido acetilsalicílico, e três (25%) corticosteroides. Ácido acetilsalicílico foi contraindicado em três dos 12 pacientes com inflamação da artéria coronária, devido à trombocitopenia e/ou úlcera péptica. Nenhum paciente teve normalização do z-score das artérias coronárias durante o seguimento.

Quatro pacientes apresentaram regurgitação tricúspide e mitral leve, e um paciente regurgitação aórtica leve. Todos esses apresentavam SIM-P.

Quatro (8,3%) pacientes apresentaram HP leve, o que foi associada à SIM-P: quatro (20%) x 0 (0%); p = 0.0025.

Oito (16,6%) pacientes apresentaram discreta efusão pericárdica transitória, que foi associada com SIM-P: oito (40%) x 0 (0%); p = 0,0003. Cinco desses apresentaram disfunção sistólica do ventrículo esquerdo, dois apresentaram disfunção sistólica do VD, e somente um HP.

### Variabilidade intraobservador e entre observadores das medidas ecocardiográficas

A reprodutibilidade das medidas de FEVE, TAPSE e artérias coronárias foi considerada boa, como demonstrada pelo CCI ≥ 0,85 para a variabilidade intraobservador e entre observadores. ( Tabela 4 ).

**Tabela 4:** – Reprodutibilidade para fração de ejeção do ventrículo esquerdo, excursão sistólica do plano anular tricúspide (TAPSE) e diâmetro interno das artérias coronárias

Parâmetro	Viés	limite de concordância de 95%	ICC
**Variabilidade intraobservador**			
FEVE (%)	0,2	-1,82 a 2,22	1
TAPSE (cm)	-0,09	-0,42 a 0,24	0,92
ACE (mm)	0	-0,01 a 0,02	0,9
ADE (mm)	0	-0,01 a 0,01	1
AC (mm)	0	-0,02 a 0,01	0,95
ACD (mm)	0	-0,01 a 0,01	0,98
**Variabilidade entre observadores**			
FEVE (%)	0,4	-3,95 a 4,75	0,98
TAPSE (cm)	-0,08	-0,49 a 0,33	0,85
ACE (mm)	0,01	-0,01 a 0,02	0,9
ADE (mm)	0	-0,02 a 0,02	1
AC (mm)	0	-0,02 a 0,03	0,99
ACD (mm)	0	-0,02 a 0,01	0,98

*FEVE: fração de ejeção do ventrículo esquerdo; TAPSE: excursão sistólica do plano anular tricúspide; ACE: artéria coronária esquerda; ADE: artéria descendente anterior esquerda; AC: artéria circunflexa; ACD: artéria coronária direita.*

## Discussão

O presente estudo destaca-se pela avaliação sistemática de achados ecocardiográficos de uma coorte de pacientes pediátricos com COVID-19, com alta prevalência de doenças pré-existentes. Associações significativas de anormalidades cardíacas com parâmetros clínicos, laboratoriais e terapêuticos foram claramente demonstrados, reforçando o papel importante do acompanhamento ecocardiográfico dessa população.

Estudos publicados desde abril de 2020, conduzidos no Reino Unido, na França, Itália, Suíça e América do Norte relataram que a SIM-P é temporariamente relacionada à SARS-CoV-2, e frequentemente associada à tempestade de citocina, disfunção cardiovascular grave, admissão à UTI pediátrica, e morte.^16^ Diferentemente do presente estudo, a maioria das crianças e adolescentes nesses estudos não apresentavam comorbidades, o que pode ter contribuído para os melhores desfechos. Enquanto Feldstein et al.^17^ relataram 2% de mortes em uma população em que 73% eram indivíduos previamente sadios, este estudo mostrou uma taxa de mortalidade de 14,6% em uma população com 27% de indivíduos sadios. Similar à maioria dos estudos publicados, os pacientes com SIM-P no presente estudo apresentaram menos testes de RT-PCR positivos que os pacientes sem SIM-P, sugerindo que essa síndrome é um fenômeno pós-infeccioso relacionado a uma resposta imune exacerbada que ocorre algumas semanas após a fase aguda. Somente um paciente apresentou-se com sinais de inflamação mucocutânea, reforçando que a SIM-P e a doença de Kawasaki são doenças realmente diferentes, que compartilham algumas características clínicas.^3^

O maior estudo ecocardiográfico global já publicado revelou presença de anormalidades cardíacas e, 46% dos adultos com COVID-19 sem doenças cardíacas pré-existentes. O presente estudo descreve, pela primeira vez, anormalidades ecocardiográficas em 39,6% das crianças com COVID-19 avaliadas, potencialmente relacionadas à infecção por SARS-CoV-2.^18^

A maioria dos estudos relatando anormalidades cardíacas em pacientes pediátricos com COVID-19 envolveu muitos centros, sem um protocolo comum de avaliação ecocardiográfica. Até o momento, em uma das maiores séries publicadas que incluiu 186 pacientes com SIM-P de 26 estados dos Estados Unidos, a FEVE foi avaliada quantitativamente ou qualitativamente.^17^ A padronização dos métodos ecocardiográficos e a inclusão de testes de variabilidade intraobservador e entre observadores neste estudo pode ter contribuído para uma estimativa mais confiável da incidência de anormalidades cardíacas em crianças com COVID-19. Por exemplo, foi detectado duas vezes mais disfunção sistólica do ventrículo esquerdo em pacientes com SIM-P (40%) em comparação ao estudo de Feldstein et al. (20%).^17^

Apesar de a disfunção sistólica ventricular em pacientes pediátricos com COVID-19 ter sido descrita extensivamente, os mecanismos fisiopatológicos envolvidos na lesão do miocárdio foram pouco investigados.^19 , 20^ Partículas virais foram observadas no miocárdio e no endotélio vascular em pacientes adultos com COVID-19 e choque cardiogênico.^21 , 22^ Ainda, autópsias mostraram infiltrados inflamatórios compostos por macrófagos, CD4+, e células T, associados com regiões de necrose de cardiomiócitos. Ainda não está claro quanto da lesão cardíaca pode ser diretamente atribuída à infecção viral versus resposta inflamatória sistêmica.^1^ Apesar dos mecanismos envolvidos, pacientes adultos com níveis elevados de biomarcadores de lesão do miocárdio (troponina, pró-BNP) estão em risco significativamente maior de morte.^23^ No presente estudo, observamos níveis maiores de troponina nos pacientes com disfunção de ventrículo esquerdo e direito, o que destaca uma possível contribuição da disfunção cardíaca a piores desfechos nos pacientes pediátricos com COVID-19. De fato, pacientes com anormalidades ecocardiográficas necessitaram de um suporte ventilatório e de drogas vasoativas mais agressivo, maior tempo de internação, e apresentaram uma maior taxa de mortalidade que aqueles com ecocardiogramas normais.

Além disso, sabe-se que níveis séricos de marcadores inflamatórios, tais como ferritina e PCR, são mais elevados em pacientes com COVID-19 que sobreviveram que aqueles que foram a óbito. Tal fato reflete os efeitos deletérios da resposta inflamatória difusa em múltiplos órgãos, incluindo o coração.^24^ Níveis mais altos de ferritina sérica e PCR foram detectados em pacientes com disfunção sistólica do ventrículo esquerdo em nosso estudo, o que pode ter contribuído um baixo débito cardíaco, hipoperfusão tecidual, e disfunção de múltiplos órgãos.

Vale enfatizar que nenhum paciente recebeu bloqueadores de interleucina durante o período do estudo, uma vez que naquele tempo, ainda existia pouca informação sobre seu uso em pacientes pediátricos com COVID-19.

Outro mecanismo importante de dano do miocárdio que deve ser destacado é a lesão microvascular, com formação de microtrombos na vasculatura do miocárdio e consequente isquemia.^1^ Recentemente, Duarte-Neto et al.^25^ identificaram pequenos trombos nos vasos do miocárdio utilizando autópsia minimamente invasiva guiada por ultrassom em adultos com COVID-19. Isso pode explicar por que níveis mais elevados de d-dímero foram observados em pacientes com disfunção sistólica do VD e ventrículo esquerdo. Diretrizes recentes em pacientes pediátricos com COVID-19 ainda não recomendam anticoagulação profilática para todos os pacientes com SIM-P. Ainda, a terapia de anticoagulação é restrita a pacientes com FEVE<30% ou com aneurismas gigantes de artéria coronária (z-score da artéria coronária ≥+10).^26^ Os achados do presente estudo sugerem um possível benefício em se administrar, de maneira profilática, heparina de baixo peso molecular a pacientes com SIM-P para prevenir isquemia do miocárdio e disfunção ventricular. São necessários estudos prospectivos com um número maior de pacientes pediátricos para confirmar nossa hipótese.

Foi detectada uma alta incidência (25%) de anormalidades da artéria coronária nos pacientes estudados. De fato, a extensão do acometimento das artérias coronárias em crianças com COVID-19 ainda é motivo de preocupação. Enquanto alguns autores descreveram uma taxa de 14% dos pacientes com SIM-P que apresentam dilatação de artéria coronária,^19^ outros relataram uma taxa de 41% de artérias coronárias ecogênicas e proeminentes na admissão, apesar de diâmetros normais.^20^ Essas discrepâncias provavelmente refletem diferentes protocolos de avaliação: em alguns estudos, somente os z-scores da artéria coronária foram considerados, enquanto em outros, sinais precoces de inflamação da artéria coronária também foram incluídos (como realce perivascular e ausência de afilamento). Ainda, o delineamento longitudinal do presente estudo pode ter viabilizado uma detecção mais precisa de anormalidades das artérias coronárias, uma vez que 33,3% dos pacientes foram submetidos aos exames de imagem várias vezes durante a internação. De fato, um terço dos pacientes não apresentaram anormalidades das artérias coronárias no primeiro exame, somente em exames subsequentes.

Anormalidades coronárias na COVID-19 foram recentemente associadas à tempestade de citocinas na SIM-P, especialmente interleucna-6.^27^ Os níveis mais altos de d-dímero nos pacientes com dilatação de artéria coronária em nosso estudo destacam uma importante via fisiopatológica que necessita ser mais investigada em pacientes com SIM-P. Com base no papel crescente da imunotrombose nas doenças pediátricas, tais como sepse e doenças reumáticas autoimunes, pode-se levantar a hipótese de que bloquear a cascata de coagulação pode contribuir para diminuir a resposta inflamatória.^28^ De fato, várias publicações descreveram as propriedades não anticoagulantes da heparina, tais como inibindo a quimiotaxia de neutrófilos e a migração de leucócitos, neutralizando o fator de complemento C5a carregado positivamente, e sequestrando proteínas de fase aguda, com consequente diminuição de biomarcadores inflamatórios.^29^ Portanto, a heparina também atuaria como terapia anti-inflamatória adjuvante em pacientes com SIM-P e inflamação da artéria coronária, juntamente com imunoglobulina endovenosa, corticosteroides e agentes imunobiológicos.

### Limitações

O presente estudo tem limitações dado seu caráter retrospectivo, embora as avaliações ecocardiográficas tenham sido padronizadas e a variabilidade intraobservador e entre observadores tenha sido adequada. O grupo de estudo foi formado predominantemente por pacientes com doenças pré-existentes, o que pode dificultar extrapolações dos resultados para crianças previamente sadias. Por exemplo, a notável prevalência de malignidades na população avaliada pode ter contribuído para o estado de hipercoagulação, e a disfunção ventricular subclínica. Ainda, pacientes com doença renal crônica ou doenças reumatológicas eram mais propensos à inflamação, não necessariamente causada por COVID-19. Finalmente, imunossupressão pode ter contribuído para a baixa frequência de sorologia positiva para SARS-CoV-2 em nossos pacientes com SIM-P.

## Conclusões

Anormalidades ecocardiográficas em pacientes pediátricos com COVID-19 são frequentes e associadas a piores desfechos clínicos. Associações entre anormalidades ecocardiográficas e biomarcadores de inflamação/coagulação revelaram possíveis vias fisiopatológicas para explicar a presença de lesão do miocárdio em nossos pacientes pediátricos com COVID-19. Mais estudos devem ser conduzidos para determinar quais estratégias terapêuticas irão reduzir a disfunção cardiovascular nessa população, considerando os diferentes mecanismos de lesão miocárdica. A ecocardiografia está bem posicionada para ajudar nesse entendimento, como uma tecnologia barata, portátil e de amplo acesso.
