# Supplementary material for: O Coração de Pacientes Pediátricos com COVID-19: Novos Insights a Partir de um Estudo Ecocardiográfico Sistemático em um Hospital Terciário no Brasil
Source: Arq Bras Cardiol. 2021 Jun 10;117(5):954–64. [Article in Portuguese] doi: 10.36660/abc.20200920 (PMC8682092; doi:10.36660/abc.20200920)
Supplement: Translation

# Translation: The Heart of Pediatric Patients with COVID-19: New Insights from a Systematic Echocardiographic Study in a Tertiary Hospital in Brazil

**Authors:** Maria de Fátima Rodrigues Diniz, Maira Freire Cardoso, Karen Saori Shiraishi Sawamura, Carolina Rocha Brito Menezes, Alessandro Cavalcanti Lianza, Maria Fernanda Badue Pereira, Nadia Litvinov, Juliana Ferreira Ferranti, Silvana Forsait, Andreia Watanabe, Sylvia Costa Lima Farhat, Nadia Emi Aikawa, Lucia Maria Arruda Campos, Artur Figueiredo Delgado, Magda Carneiro-Sampaio, Werther Brunow de Carvalho, Clovis Artur Silva, Gabriela Nunes Leal

**Affiliations:** 1 Brazil Universidade de São Paulo Instituto da Criança, São Paulo, SP - Brazil; 2 Brazil Hospital do Coração, São Paulo, SP - Brazil; 3 Brazil Hospital Israelita Albert Einstein, São Paulo, SP - Brazil; 4 Brazil Universidade de São Paulo Hospital das Clínicas - Faculdade de Medicina, São Paulo, SP - Brazil; 5 Brazil Universidade de São Paulo - Faculdade de Medicina, São Paulo, SP - Brazil; 6 Brazil Hospital Sírio-Libanês, São Paulo, SP - Brazil

**Keywords:** COVID-19, Pandemics, Betacoronavirus, Biomarkers, Inflammation, Child, Heart Failure, Echocardiography/methods

## Abstract

**Background:**

COVID-19 pandemic represents a huge burden to the health system in the world. Although pediatric COVID-19 patients have been relatively spared compared with adults, recent reports showed an increasing number of critically ill patients with multisystemic inflammatory syndrome in children (MIS-c), with marked cardiovascular impairment. Nevertheless, little is known about the relationship between cardiac abnormalities and inflammatory and coagulation biomarkers.

**Objectives:**

to investigate echocardiographic abnormalities in pediatric patients with COVID-19 admitted to tertiary hospital.

**Methods:**

this was a retrospective longitudinal study, based on the review of medical records and echocardiograms of patients (0-19 years) admitted to a tertiary hospital between March 30 and June 30, 2020. For statistical analysis, the significance level was set at 5% (p < 0.05).

**Results:**

Forty-eight patients were enrolled, 73% with preexisting diseases, 20 (41.7%) with MIS-c. Median age was 7.5 (0-18.6) years; 27 (56.2%) were male. Median duration of hospitalization was 15.4 (2-92) days and seven (14.6%) patients died. A total of 70 echocardiograms were performed; 66.7% patients were scanned only once and 33.3% multiple times. Twenty-three (48%) patients showed echocardiographic abnormalities: eight (16.6%) left ventricle (LV) systolic dysfunction, six (12.5%) right ventricle (RV) systolic dysfunction and 12 (25%) coronary dilatation (Z-score>+2.5). Echocardiographic abnormalities were significantly associated with MIS-c, admission to the pediatric intensive care unit, multiple organ dysfunction, ventilatory/vasoactive support, and death (p<0.05). Significantly higher d-dimer (ng/mL) levels were detected in patients with LV dysfunction [16733(4157-115668) vs. 2406.5(190-95040)], RV dysfunction [25769(3422-115668) vs. 2803.5(190-95040)] and coronary artery dilation [9652.5(921-115668) vs. 2724(190- 95040)] (p<0.05).

**Conclusion:**

Echocardiographic abnormalities in COVID-19 pediatric patients were frequent and associated with worse clinical outcomes. Exacerbation of the inflammation and coagulation pathways may play an important role in cardiovascular injury in those patients.

## Introduction

The coronavirus disease 19 (COVID-19) pandemic represents a huge burden to the health system in the world. In its severe presentation, COVID-19 is a systemic illness characterized by hyperinflammation, cytokine storm and elevated myocardial injury markers.^1^ Cardiac involvement appears to be a prominent feature of the disease in adults, occurring in 20% to 30% of hospitalized patients and contributing to 40% of deaths.^2^ Although children have been relatively spared compared to adults, recent reports showed an increasing number of critically ill children with multisystem inflammatory syndrome (MIS-C, multisystem inflammatory syndrome in children), accompanied by severe cardiovascular impairment.^3^ Ventricular dysfunction, pericardial effusion, valvar regurgitation and coronary artery inflammation were documented in many case series. A Kawasaki-like phenotype was also described in some MIS-C patients, although recent literature suggests these are two different illnesses with overlapping clinical features. To date, MIS-C occurs predominately in older children, with a median age of 9-10 years, whereas Kawasaki disease typically affects patients younger than five years. Cardiovascular shock, rarely seen in Kawasaki disease, is a striking feature of MIS-C.^3^

Nevertheless, the real incidence of overall cardiac abnormalities among pediatric COVID-19 patients and their relevance to clinical outcomes are yet to be determined. Little is known about the relationship between cardiac abnormalities, and inflammatory and coagulation markers in this group.^4^ Consequently, there is an urgent need to better understand the interactions between COVID-19 and the heart in the pediatric population.

The present study aimed to investigate echocardiographic abnormalities of pediatric COVID-19 patients admitted to a tertiary hospital in São Paulo, the epicenter of coronavirus pandemic in Brazil. Possible associations of clinical and laboratory data with echocardiographic findings were also explored.

## Methods

### Study design and population

This is a longitudinal retrospective study, based on the review of medical records and echocardiogram reports from children and adolescents (0-19 years) admitted to the pediatric ward and intensive care unit due to COVID-19, between March 30 and June 30, 2020. Patients with and without MIS-c were included, according to the World Health Organization (WHO) classification.^5^ Exclusion criterion was the absence of echocardiograms during the follow-up period.

### Clinical, laboratory and therapeutic parameters

Patients’ electronic medical records were carefully reviewed for clinical, laboratory and therapeutic data. Pre-existing diseases and previous echocardiogram reports were also registered. The Institutional Research Ethics Committee has approved the study.

Patients were classified as having MIS-C if they fulfilled the following criteria:

1. Children and adolescents (0-19 years) with fever for three or more days;
2. And at least two of the following:
  - Rash, bilateral non-purulent conjunctivitis, or mucocutaneous inflammation signs (oral, hands or feet).
  - Hypotension or shock
  - Features of myocardial dysfunction, pericarditis, valvulitis, or coronary abnormalities (including echocardiographic findings or elevated cardiac enzymes).
  - Evidence of coagulopathy (by elevated d-dimers, prothrombin time, partial thromboplastin time)
  - Acute gastrointestinal problems (diarrhea, vomiting or abdominal pain).
3. And: elevated inflammatory markers such as erythrocyte sedimentation rate (ESR), C-reactive protein (CRP) or procalcitonin.
4. And: no other obvious microbial cause of inflammation
5. And: severe acute respiratory syndrome coronavirus 2 (SARS-CoV-2) confirmed infection by real time-polymerase chain reaction (RT-PCR) and/or serology, or likely contact with patients with COVID-19.

RT-PCR in respiratory specimens were performed to detect SARS-CoV-2 RNA. Serologic tests included two different methods during the COVID-19 pandemic: immunochromatography assay for SARS-Cov-2 specific IgM/IgG antibody detection and anti-SARS-CoV-2 enzyme-linked immunosorbent assay (ELISA) for IgG antibodies detection.^6^

MIS-C and non-MIS-C patients were compared regarding age, sex, clinical signs and symptoms at presentation, frequency of echocardiographic abnormalities, confirmed SARS-CoV-2 infection and death. The following laboratory data were compared: frequency of anemia, lymphocytopenia and thrombocytopenia, evidence of coagulopathy, peak levels of d-dimer, CRP, ferritin, troponin, and creatine kinase-MB. Pro-brain natriuretic peptide (BNP), procalcitonin and fibrinogen were not included in the analysis, because these biomarkers were not assessed routinely in all patients.

Anemia was defined as hematocrit at or below the 2.5^th^percentile for age, race and gender;^7^ lymphocytopenia was defined as a lymphocyte count was lower than 4,500/mm^3^in children under eight months of age and 1500/mm^3^in older ones;^8^ and thrombocytopenia was defined as platelet count lower than 100,000/microL.^9^

### Echocardiography

All echocardiographic tests were performed by two experienced pediatric cardiologists, according to the guidelines of the American Society of Echocardiography (ASE).^10^ The analyses included M and two-dimensional (2D) modes, besides standard Doppler examinations with color flow mapping. The equipment used was a Philips Affinity 70, CX50 and Innosight compact ultrasound, with multi-frequency transducers (S5-1 and S8-3). Echocardiographic studies also followed the ASE statement on protection of patients and echocardiography service providers during COVID-19 outbreak.^11^ Since one of the equipment used in our institution during the COVID-19 pandemic was originally designed as a point-of-care ultrasound (Philips Innosight), it was not possible to obtain 2D derived left ventricular ejection fraction (LVEF) (Simpson’s method) in all scans. For that reason, M-mode derived LVEF (Teichholz method) was chosen for this study purpose, although Simpson’s method would be undoubtedly more accurate.^10^ Left ventricular (LV) systolic dysfunction was defined as a LVEF lower than 55%; it was considered mild if the LVEF was ≥ 45% and < 55%, moderate if the LVEF was ≥ 30% and < 45%, and severe if the LVEF was < 30%.^10^

Right ventricular (RV) systolic function was evaluated by tricuspid annular plane systolic excursion (TAPSE). RV systolic dysfunction was detected when TAPSE z-score was lower than -2.^12^

Coronary arteries were evaluated according to the American Heart Association Statement for Diagnosis, Treatment and Long-Term Management of Kawasaki Disease.^13^ Dilation was detected when the coronary artery internal lumen diameter z-score was higher than higher than +2.5.^14^ A z-score between +2.5 and +5 defined small aneurisms; between +5 and +10, medium aneurisms; equal or greater than +10, giant aneurisms. Other echocardiographic signs frequently described in coronary artery inflammation, like enhanced perivascular brightness and lack of tapering, were also registered.^13^

Pulmonary artery systolic pressure (PASP) was estimated through tricuspid regurgitation; pulmonary hypertension (PH) was diagnosed when the pulmonary artery systolic pressure was greater than 35 mmHg. Mild PH was diagnosed if 35 mmHg < PASP ≤ 45 mmHg, moderate if 45 mmHg < PASP ≤ 50 mmHg and severe if PASP > 50 mmHg.^15^

Pericardial effusion presence was also described, as well as eventual signs of pericardial tamponade.

Patients were divided according to the presence or absence of echocardiographic abnormalities and compared regarding age, gender, presence of MIS-c, pediatric intensive care unit (PICU) admission, presence of multiple organ dysfunction, ventilatory/vasoactive support, renal replacement therapy, use of intravenous (IV) immunoglobulin, corticosteroids, acetylsalicylic acid and low molecular weight heparin, length of hospital stay, and death.

Images were digitally acquired, and intra and interobserver variability of LVEF, TAPSE and coronary arteries diameter was evaluated. The same examiner repeated analysis of 10 randomly selected exams. A second observer (CRB), unaware of previous results and of patient’s clinical condition, also performed offline echocardiographic measures.

### Statistical analysis

Statistical analysis was performed using IBM SPSS Statistics 22. Categorical data were reported as percentages and continuous data as mean (standard deviation - SD) or median (range). Fisher exact test was chosen to compare categorical data. The Kolmogorov and Smirnov test was used to verify if data were sampled from a Gaussian distribution. Unpaired Student’s t test was used to assess continuous data with normal distribution, and the Mann-Whitney U test to assess continuous data without normal distribution. The significance level was set at 5% (p < 0.05). Intra and interobserver variability for echocardiographic measures was assessed using Bland Altman Plot and intraclass correlation coefficient (ICC), with good correlation being defined as ICC > 0.8.

## Results

### Clinical presentation

Forty-eight pediatric patients were hospitalized due to COVID-19 during the study period. The median age was 7.5 (0 - 18.6) years; 21 (43.8%) were female. The median in-hospital stay was 15.4 (2 - 92) days. By the end of the study, 33 (68.7%) patients have been successfully discharged, eight (16.7%) were still in the pediatric ward or PICU, and seven (14.6%) patients died. All deaths occurred in the MIS-c group. There was no statistically significant difference between survivors and deceased patients regarding the use of corticosteroids, IV immunoglobulin or low molecular weight heparin.

Twenty (41.7%) patients fulfilled the WHO criteria for MIS-c and 28 (58.3%) did not. Among MIS-c patients, 11 (55%) had confirmed SARS-CoV-2 infection by RT-PCR and/or serology and nine (45%) patients did not. All nine MIS-c patients without confirmed SARS-CoV-2 infection have had intimate contact with COVID-19 patients in the last four weeks before symptoms. Five of those nine patients had also typical findings on chest computed tomography (ground-glass opacities with surrounding consolidation (halo sign).

In the non-MIS-c patients (n=28), the SARS-CoV-2 infection was confirmed by PCR and/or serology.

MIS-c was associated with seizures, shock, evidence of coagulopathy, echocardiographic abnormalities, and death. Significantly higher peak values of serum d-dimer, CRP and troponin were detected among MIS-c patients (p<0.05). The incidence of respiratory and gastrointestinal symptoms was similar between MIS-c and non-MISC-c patients (p>0.05). Likewise, there was no difference between the frequency of anemia, lymphocytopenia, thrombocytopenia or preexisting diseases between the two groups of patients (p>0.05). Only one patient had with mucocutaneous inflammation signs. No patient fulfilled the diagnostic criteria for Kawasaki disease ( Table 1 ).

**Table 1:** – Demographic data, clinical and laboratory data of COVID-19 pediatric patients with and without multisystem inflammatory syndrome in children (MIS-C), according to the World Health Organization criteria (WHO)

Demographic, clinical and laboratory data	MIS-C ( *n* = 20)	Non-MIS-C ( *n* = 28)	p
Age (years)	8.4 (0.1-16.4)	6.7 (0 – 18.6)	0.33
Sex (male)	10 (50%)	17 (60.7%)	0.56
Preexisting diseases	15 (75%)	20 (71.4%)	1
Respiratory symptoms	10 (50%)	14 (50%)	1
Gastrointestinal symptoms	6 (30%)	7 (25%)	0.75
Rash/ Non-purulent bilateral conjunctivitis/mucocutaneous inflammation signs	1 (5%)	0 (0%)	0.41
Seizures	5 (25%)	0 (0%)	0.009
Shock	12 (60%)	0 (0%)	<0.0001
Evidence of coagulopathy (↑PT, ↑PTT, ↑D-dimer)	20 (100%)	18 (64.3%)	0.0028
Anemia*	14 (70%)	21 (75%)	0.75
Thrombocytopenia**	4 (20%)	7 (25%)	0.74
Lymphocytopenia***	8 (40%)	14 (50%)	0.56
Echocardiographic abnormalities	19 (95%)	4 (14.3%)	<0.0001
D-dimers (ng/ml)****	9652.5 (921 - 115668)	1722 (190 - 95040)	0.0003
C-reactive protein (mg/L)****	119.6 (0.38 - 447.7)	14.6 (0.30 – 324)	0.0046
Ferritin (ng/ml)****	1159 (58-35967)	655 (25-2567)	0.07
Troponin (ng/L)****	25 (9-385)	16 (3-1050)	0.028
Creatine kinase (CK-MB) (ng/ml)****	1.78 (0.3-30)	1.65 (0.18-28.9)	1
Death	7 (35%)	0 (0%)	0.001
Confirmation of Sars-CoV-2 infection (RT-PCR/serology)	11 (55%)	28 (100%)	0.0001

Values are expressed as n (%) or as median (range). Fisher exact test was used to compare categorical data. Mann-Whitney U test was used to compare non-normally distributed continuous variables. *Hematocrit at or below 2.5^th^percentile for age, race, and sex at admission. **Platelet count < 100000/microL at admission; ***Lymphocyte count < 4,500/mm^3^in children under 8 months and < 1500/mm^3^in older ones at admission; ****Values correspond to the highest serum level obtained from each patient. PT: prothrombin time; PTT: partial thromboplastin time

Preexisting diseases were detected in 35 (73%) patients: immunosuppression in 26 (54.2%), malignancies in 14 (40%), chronic kidney disease in nine (25.7%), chronic neuropathy in eight (22.8%), congenital/acquired heart disease in five (14.2%), chronic pneumopathy in five (14.2%), hepatopathy in four (11.4%), dysmorphic syndromes in three (6.3%), Duchenne muscular dystrophy in one (2.8%), juvenile systemic lupus erythematosus in one (2.8%), previous orthopedic surgery in 1(2.8%), previous gynecologic surgery in one (2.8%) and previous heart transplantation in one (2.8%). Two (4.2%) patients were neonates born to COVID-19 mothers and one of them was also preterm. Among the seven deceased patients, three were oncologic (two solid tumors and one leukemia), one had primary immunodeficiency, one had Edwards syndrome with congenital heart disease and two were healthy.

### Echocardiographic evaluation

The 48 patients had at least one echocardiogram performed during hospitalization. Thirty-two patients (66.7%) had only one exam and 16 (33.3%) were scanned multiple times. A total of 70 exams were carried out throughout the study. All patients with preexisting diseases were already followed at our institution and had previous echocardiogram reports in their medical records. Five (14.2%) patients had previous echocardiographic abnormalities: one with small ventricular septal defect and bicuspid aortic valve (Edward’s syndrome), one with small residual ventricular septal defect and discrete aortic coarctation, one with echogenic mass invading inferior vena cava (adrenal tumor), one with discrete left ventricle hypertrophy secondary to chronic kidney disease and one with moderate left ventricle systolic dysfunction secondary to chemotherapy (sarcoma).

Twenty-three (48%) patients had echocardiographic abnormalities and 19 (39.6%) of them showed new echocardiographic findings potentially associated with COVID-19 as follows: left and RV systolic dysfunction, coronary dilation, PH, and pericardial effusion. Of note, only one patient with previous echocardiographic abnormalities presented with new echocardiographic findings: LV systolic dysfunction secondary to chemotherapy progressed from moderate to severe and coronary dilation was also detected.

Echocardiographic abnormalities were associated with MIS-c, PICU admission, multiple organ dysfunction, ventilatory and vasoactive support, use of IV immunoglobulin, corticosteroid, acetylsalicylic acid and low molecular weight heparin, and death ( Table 2 ). Patients with echocardiographic abnormalities also had significantly higher length of hospital stay.

**Table 2:** – Demographic data e clinical outcomes according to the presence or absence of echocardiographic abnormalities

Demographic data, treatment strategies and clinical outcomes	Echocardiographic abnormalities		
	Present ( *n* = 23)	Absent ( *n* = 25)	p
Age (years)	7.8 (0.1-16.4)	6.4 (0-18.6)	0.87
Sex (male)	11 (47.8%)	16 (64%)	0.38
MIS-c according to WHO criteria	19 (82.6%)	1 (4%)	<0.0001
Pediatric Intensive Care Unit	15 (65.2%)	5 (20%)	0.003
Multiple organ dysfunction syndrome	8 (34.8%)	0 (0%)	0.0013
Respiratory system	6 (26%)	0 (0%)	
Cardiovascular system	6 (26%)	0 (0%)	
Renal system	5 (21.7%)	0 (0%)	
Hepatic system	2 (8.7%)	0 (0%)	
Neurological system	4 (17.4%)	0 (0%)	
Hematological system	4 (17.4%)	0 (0%)	
Ventilatory support	15 (65.2%)	7 (28%)	0.02
Oxygen by nasal catheter	8 (34.8%)	3 (12%)	
Venturi mask	3 (13%)	1 (4%)	
Non-rebreather mask	0 (0%)	1 (4%)	
High-flow oxygen therapy	6 (26%)	1 (4%)	
Noninvasive ventilation	5 (21.7%)	1 (4%)	
Conventional Mechanical ventilation	10 (43.5%)	3 (12%)	
High-frequency ventilation	1 (4.3%)	0 (0%)	
Vasoactive drug support	10 (43.5%)	1 (4%)	0.0015
Epinephrine	4 (17.4%)	0 (0%)	
Norepinephrine	10 (43.5%)	1 (4%)	
Vasopressin	2 (8.7%)	0 (0%)	
Milrinone	5 (21.7%)	1 (4%)	
Dobutamine	3 (13%)	0 (0%)	
Renal replacement therapy	5 (21.7%)	2 (8%)	0.23
Peritoneal dialysis	0 (0%)	2 (8%)	
Conventional hemodialysis	1(4.3%)	0 (0%)	
Sustained low-efficiency dialysis	1 (4.3%)	0 (0%)	
Continuous hemodialysis	3 (13%)	0 (0%)	
IV Immunoglobulin	14 (60.8%)	0 (0%)	<0.0001
Corticosteroids	4 (17.4%)	0 (0%)	0.04
Acetylsalicylic acid	9 (39%)	0 (0%)	0.0005
Low molecular weight heparin	9 (39.1%)	1 (4%)	0.0038
Length of stay (days)	23 (2-92)	8.3 (2-26)	0.0074
Deaths	6 (26%)	1 (4%)	0.04

*Values are expressed as n (%) or as median (range). Fisher exact test was used to compare categorical data. Mann-Whitney U test was used to compare non-normally distributed continuous variables.*

Ten (20.8%) of the 48 patients received low molecular weight heparin during hospitalization, only one patient without echocardiographic abnormalities. Therapeutic anticoagulation was introduced in two patients: one with left main coronary artery (LMCA) z-score of +10 and one with LV severe systolic dysfunction and subclavian vein thrombosis. The remaining eight patients received prophylactic low molecular weight heparin due to prolonged hospitalization, concomitant malignancies, and prolonged catheter use.

LV systolic dysfunction was detected in eight (16.6%) patients: six with mild, one with moderate and one with severe dysfunction. Global hypokinesia of left ventricle was found in all but one patient, who exhibited apical akinesia suggestive of Takotsubo syndrome. Four patients with LV systolic dysfunction had also coronary arteries abnormalities. Patients with LV systolic dysfunction showed significantly higher peak levels of d-dimer, CRP, ferritin, and troponin ( Table 3 ). Five patients with LV systolic dysfunction received IV immunoglobulin and only one received corticosteroids. No patient received interleukin blockers. Five patients showed improvement of LV systolic function during the follow-up.

**Table 3:** – Laboratorial profile of patients according to the echocardiographic abnormalities detected throughout the study

Laboratory*	LV systolic dysfunction			RV systolic dysfunction			Coronary artery abnormalities		
	Present (n = 8)	Absent (n = 40)	p	Present (n = 6)	Absent (n = 42)	p	Present (n =12)	Absent (n = 36)	p
D-dimers (ng/ml)	16733 (4157 -115668)	2406.5 (190 - 95040)	0.0015	25769 (3422 - 115668)	2803.5 (190 - 95040)	0.037	9652.5 (921 - 115668)	2724 (190 - 95040)	0.04
CRP (mg/L)	303.16 (30 - 423)	35.9 (0.3 - 447.7)	0.0017	113.95 (2 - 407.21)	53.95 (0.3 - 447.70)	0.46	109.9 (0.38 – 423)	33.75 (0.38 - 447)	0.10
Ferritin (ng/ml)	3734 (839 - 35967)	499 (25 - 8000)	0.0026	1301 (123 - 35967)	663 (25 - 8000)	0.18	389.50 (58 - 35967)	790 (25 - 8000)	0.8
Troponin (ng/L)	88 (20 - 342)	16 (3 - 1050)	0.0018	108.5 (3 - 385)	17 (3 - 1050)	0.04	19.5 (9 - 125)	19 (3 - 1050)	0.57
CK-MB (ng/ml)	2.2 (0.7 - 28)	1.6 (0.18 - 30.7)	0.62	4 (0.18 - 30.7)	1.6 (0.3 -28.9)	0.58	1.78 (0.3 -18.2)	1.65 (0.18 – 30.7)	0.9

Mann-Whitney U test was used to compare non-normally distributed continuous variables. *Values correspond to the highest level obtained from each patient and are expressed as median (range).

Six (12.5%) patients showed RV systolic dysfunction. These patients showed significantly higher peak levels of d-dimer and troponin ( Table 3 ). Two patients had mild PH and three had also LV systolic dysfunction. RV systolic function improved in three patients during follow-up.

Coronary abnormalities were detected in 12 (25%) patients and most of them exhibited mild ectasia, except for an adolescent (15 years old) with a LMCA z-score of +10 ( Figure1 ). Besides dilation, six patients had also enhanced perivascular brightness. Dilatation of the LMCA was observed in 11 patients, with a median z-score of +4 (+2.8 - +10); dilation of the left descending coronary artery (LDCA) in six, with a median z-score of +4 (+3.6 - +4.2); dilatation of the left circumflex coronary artery (LCCA) in three, with a median z-score of +4.6 (+3.9 - +5); and dilatation of the right coronary artery (RCA) in four, with a median z-score of +3.3 (+2.6 - +4.3). Patients with coronary arteries abnormalities had significantly higher peak levels of d-dimers ( Table 3 ). In four patients, coronary abnormalities were not present in the first echocardiographic evaluation and were detected in subsequent scans.

**Figure 1:** – A) Echocardiogram showing dilated left main coronary artery (LMCA) in a 15-year old girl; B) Computed tomography of the same patient.

Eleven (91.7%) of the 12 patients with coronary dilatation received IV immunoglobulin. One had coronary dilatation detected belatedly, after being afebrile for more than one week. Nine (75%) of the 12 patients with coronary dilatation received acetylsalicylic acid and three (25%) corticosteroids. Acetylsalicylic acid was contraindicated in three of the 12 patients with coronary artery inflammation, due to thrombocytopenia and/or peptic ulcer. No patient exhibited normalization of coronary arteries z-score during the follow-up.

Four patients had mild tricuspid and mitral regurgitation and one patient had mild aortic regurgitation. All of them were MIS-c patients.

Four (8,3%) patients had mild PH, which was associated with MIS-c: 4 (20% with MIS-c) x 0 (0% Non-MIS-c).

Eight (16.6%) patients had discrete transient pericardial effusion, which was also associated with MIS-c: eight (40%) x 0 (0%); *p* = 0.0003. Five patients had concomitant LV systolic dysfunction, two had RV systolic dysfunction and only one PH.

### Intra- and interobserver variability of echocardiographic measures

Reproducibility of LVEF, TAPSE and coronary arteries was considered good, as demonstrated by ICC ≥ 0.85 for intra and interobserver variability ( Table 4 ).

**Table 4:** – Reproducibility for LV ejection fraction, TAPSE and coronary artery internal diameter

Parameter	Bias	95% limits of agreement	ICC
**Intraobserver variability**			
LVEF (%)	0.2	-1.82 a 2.22	1
TAPSE (cm)	-0.09	-0.42 a 0.24	0.92
LMCA (mm)	0	-0.01 a 0.02	0.9
LDCA (mm)	0	-0.01 a 0.01	1
LCCA (mm)	0	-0.02 a 0.01	0.95
RCA (mm)	0	-0.01 a 0.01	0.98
**Interobserver variability**			
LV ejection fraction (%)	0.4	-3.95 a 4.75	0.98
TAPSE (cm)	-0.08	-0.49 a 0.33	0.85
LMCA (mm)	0.01	-0.01 a 0.02	0.9
LDCA (mm)	0	-0.02 a 0.02	1
LCCA (mm)	0	-0.02 a 0.03	0.99
RCA (mm)	0	-0.02 a 0.01	0.98

*LVEF: left ventricular ejection fraction; TAPSE: tricuspid annular plane systolic excursion; LMCA: left main coronary artery; LDCA: left descending coronary artery; LCCA: left circumflex coronary artery; RCA: right coronary artery.*

## Discussion

The present study stands out for the systematic echocardiographic evaluation of a cohort of pediatric COVID-19 patients, with high prevalence of preexisting diseases. Significant associations of cardiac abnormalities with clinical, laboratorial and therapeutic parameters were clearly demonstrated, reinforcing the pivotal role of rigorous echocardiographic follow-up of this population.

Studies published since April, 2020, conducted in the UK, France, Italy, Switzerland and North America have reported that MIS-c is temporally related to SARS-CoV-2, and is frequently associated with cytokine storm, severe cardiovascular impairment, PICU admission and death.^16^ Differently from the present study, most of their children and adolescents did not have underlying comorbidities, which may have contributed to the better clinical outcomes. While Feldstein et al.^17^ reported 2% of deaths in a population where 73% were previously healthy patients, this study showed 14.6% of deaths in a population with 27% of healthy subjects. Like in the majority of published papers, the present MIS-C patients had less positive RT-PCR tests than the non-MIS-C ones, suggesting that this syndrome is a post-infectious phenomenon related to a hyperimmune response, occurring a couple of weeks after the acute phase. Only one patient presented with mucocutaneous inflammation signs, reinforcing that MIS-c and Kawasaki disease are really different illnesses sharing some clinical features.^3^

The largest global echocardiographic survey already published reveled cardiac abnormalities in 46% of adult patients with COVID-19, without preexisting cardiac diseases. Similarly, the present study documented non-previously described echocardiographic abnormalities in 39.6% of pediatric COVID-19 patients, potentially related to SARS-CoV-2 infection.^18^

Most studies reporting cardiac abnormalities in pediatric patients with COVID-19 enrolled many centers, without a common protocol of echocardiographic evaluation. To date, in one of the largest published series that included 186 patients with MIS-c from 26 states in the United States, LVEF was assessed either quantitatively or qualitatively.^17^ The standardization of echocardiographic methods and the inclusion of intra/interobserver variability tests in this study may have contributed to more reliable estimation of the incidence of cardiac abnormalities among pediatric COVID-19 patients. For instance, twice as much LV systolic dysfunction was found in MIS-c patients (40%), compared with the study of Feldstein et al. (20%).^17^

Although ventricular systolic dysfunction in pediatric patients with COVID-19 has been extensively described, the pathophysiological mechanisms involved in myocardial injury were scarcely investigated.^19 , 20^ Viral particles have been observed in the myocardium and vascular endothelium in adult patients with COVID-19 and cardiogenic shock.^21 , 22^ In addition, autopsies showed inflammatory infiltrates composed of macrophages, CD4+ and T cells, associated with regions of cardiomyocyte necrosis. It is still unclear how much of the cardiac injury can be directly attributable to viral infection versus systemic inflammatory response.^1^ In spite of the mechanism involved, adult patients with elevated biomarkers of myocardial injury (troponin, pro-BNP) are at significantly higher risk of death.^23^ In the present study, higher levels of troponin were found in patients with left and RV systolic dysfunction, highlighting the possible contribution of cardiac impairment to worse clinical outcomes in pediatric COVID-19 patients. Indeed, patients with echocardiographic abnormalities required more aggressive ventilatory and vasoactive drug support and longer hospitalization and had a higher mortality rate than those with normal echocardiograms.

Serum levels of inflammatory markers such as ferritin and CRP are also known to be higher in non-survivors than in survivors of COVID-19, reflecting deleterious effects of amplified inflammatory response to multiple organs, including the heart.^24^ Higher levels of serum ferritin and CRP were detected among patients with LV systolic dysfunction in the present study, which may have contributed to low cardiac output, tissue hypoperfusion and multiple organ dysfunction.

It is important to emphasize that no patient received interleukin-blockers during the study period, since at that time there was still limited information regarding their use in pediatric patients with COVID-19.

Another relevant mechanism of myocardial damage that must be pointed out is the microvascular injury, with microthrombi formation in the myocardial vasculature and consequent ischemia.^1^ Recently, Duarte-Neto et al.^25^ have identified small thrombi in myocardial vessels by ultrasound-guided minimally invasive autopsy in adults with COVID-19. That may explain why higher levels of d-dimer were found among patients with left and RV systolic dysfunction. Interestingly, recent guidelines regarding pediatric patients with COVID-19 still do not advocate routine prophylactic anticoagulation for all MIS-c patients. Moreover, therapeutic anticoagulation is restricted to patients with LVEF < 30% or with giant coronary aneurysms (coronary z-scores ≥ +10).^26^ The present findings suggest there may be some benefit in administrating prophylactic low molecular weight heparin to MIS-c patients, aiming to prevent myocardial ischemia and ventricular dysfunction. Prospective studies with greater number of pediatric patients will be necessary to confirm our hypothesis.

High incidence (25%) of coronary artery abnormalities was found among the patients studied. In fact, the extent of coronary artery involvement in children with COVID19 is still a matter of concern. Whereas some authors describe 14% of MIS-C patients with coronary dilatation,^19^ others have found 41% of prominent and echogenic coronary arteries on admission, despite normal diameters.^20^ These discrepancies probably reflect different protocols of evaluation: in some studies, only coronary z-scores were considered, while in others, early signs of coronary artery inflammation were also included (like enhanced perivascular brightness and lack of tapering). Moreover, the longitudinal design of the present study may have enabled more accurate detection of coronary abnormalities, since 33.3% of patients were scanned multiple times throughout hospitalization. Indeed, one third of them did not have coronary arteries abnormalities in their first scan, only in subsequent evaluations.

Coronary abnormalities in COVID-19 have been previously linked to cytokines storm in MIS-C, specially interleukine-6.^27^ The higher levels of d-dimer among patients with coronary dilation in the present study highlights an important pathophysiological pathway to be further investigated in MIS-C patients. Based on the emerging role of immunothrombosis in pediatric conditions, like sepsis and autoimmune rheumatic diseases, one can hypothesize that blocking coagulation cascade may contribute to dampen the inflammatory response.^28^ In fact, several publications have described the non-anticoagulant properties of heparin, such as inhibiting neutrophil chemotaxis and leukocyte migration, neutralizing the positively charged complement factor C5a and sequestering acute phase proteins, with a consequent decrease of inflammatory biomarkers.^29^ Thus, heparin could also work as an adjuvant anti-inflammatory therapy in MIS-c patients with coronary artery inflammation, together with IV immunoglobulin, corticosteroids and immunobiological agents.

### Limitations

The present study is limited by its retrospective nature, although echocardiographic evaluations were reasonably standardized and intra/interobserver variability was proven adequate. The study group was formed predominantly by patients with preexisting diseases, which may make extrapolation of results to previously healthy children difficult. For instance, the striking prevalence of malignancies among the referred COVID-19 patients may have contributed to the hypercoagulation state, as well as to subclinical ventricular dysfunction. Likewise, patients with chronic kidney disease or rheumatologic diseases were prone to inflammation, not necessarily caused by COVID-19. Finally, immunosuppression may have contributed to the low frequency of SARS-CoV-2 positive serology among our MIS-c patients.

## Conclusions

Echocardiographic abnormalities in pediatric patients with COVID-19 are frequent and associated with worse clinical outcomes. Associations between echocardiographic abnormalities and inflammation/coagulation biomarkers provided possible pathophysiological pathways to explain myocardial injury in our pediatric patients with COVID-19. Further studies should be conducted to determine which therapeutic strategies will reduce cardiovascular impairment in this population, taking into account the different mechanisms of myocardial damage. The follow-up of pediatric COVID-19 survivors with cardiac abnormalities will be necessary. Echocardiography is well placed to help further this understanding, as an inexpensive, portable, and widely accessible technology.
